# The relationship between peripheral T follicular helper cells and disease severity in systemic sclerosis

**DOI:** 10.1007/s10238-023-01286-9

**Published:** 2024-01-27

**Authors:** Melek Sahinoglu, Gokhan Sargin, Irfan Yavasoglu, Taskin Senturk

**Affiliations:** 1 Department of Internal Medicine, Aydin Adnan Menderes University, Aydin, Turkey; 2 Department of Rheumatology, Aydin Adnan Menderes University, Aydin, Turkey; 3 Department of Hematology, Aydin Adnan Menderes University, Aydin, Turkey

**Keywords:** Systemic sclerosis, Peripheral T follicular helper cells, Disease severity

## Abstract

We aimed to investigate the association between follicular T helper cells (Tfh) and disease severity in systemic sclerosis (SSc), a chronic connective tissue disease characterized by progressive fibrosis. While Tfh cells have been extensively studied in other autoimmune diseases, their role in SSc remains poorly understood. A cohort of 50 SSc patients, diagnosed based on the ACR/EULAR 2013 classification criteria, was included in the study. Patient data, including demographic information, comorbidities, treatment history and organ involvement, were collected. Disease severity was assessed using the modified Rodnan skin score and Medsger disease severity index. Statistical analyses were performed, considering a *p* value of < 0.05 as statistically significant. 38% had SSc with limited skin involvement, while 62% had SSc with extensive skin involvement. However, there were no statistically significant differences observed in the levels of CD4+ CXCR5+ , CD4+ ICOS+ , CD4+ CD40L+ and CD4+ PD+ lymphocytes between the two groups. Notably, SSc patients with Raynaud’s phenomenon, digital ulcer and lung involvement exhibited higher levels of CD4+ CXCR5+ lymphocytes compared to those without these manifestations. Furthermore, a significant positive correlation was observed between CD4+ CXCR5+ lymphocyte levels and the severity of lung disease according to the Medsger disease severity index. Based on these findings, we conclude that elevated levels of Tfh cells are associated with lung involvement in SSc and there is a significant correlation between Tfh cell levels and the severity of lung disease. These observations suggest a potential role for Tfh cells in the pathogenesis of lung involvement in SSc and may guide the development of targeted therapies for this aspect of the disease.

## Introduction

Systemic sclerosis (SSc) is a chronic multi-systemic connective tissue disease characterized by progressive fibrosis of the skin and/or visceral organs. The immune systems, vascular dysfunction and fibroblasts are involved in pathogenesis [1]. Perivascular infiltration is observed with activated T cells in the vascular structures in the affected tissues. Cytokines and growth factors cause endothelial activation and microvascular damage [2]. Chemoattraction of fibroblasts to the vessel wall, transdifferentiation to myofibroblasts, collagen synthesis and deposition develop with the effect of cytokines [3].

The production of autoantibodies and B cell activation is involved in the pathogenesis of most autoimmune diseases. T and B cell interactions enable the maturation of B cells and the production of pathogenic autoantibodies [4]. T cells regulate the immune response and are divided into subgroups such as Th1, Th2, Th17, Treg and Tfh [5]. Tfh cells support B cells to produce antibodies for humoral immune response [6]. Tfh cells in the germinal center contain surface molecules such as CXCR5, PD-1 and ICOS (inducible T cell co-stimulator) [7]. The interaction of surface molecules provides signals for survival, differentiation and isotype switching [8]. Junctional adhesion molecule-A (JAM-A) and JAM-C are cell adhesion molecules that have been implicated in angiogenesis, metastasis and fibrosis in various studies [9–12]. In SSc, aberrant expression of JAM-A and JAM-C has been observed in skin biopsies, dermal fibroblasts and endothelial cells [9, 10]. These molecules are believed to contribute to the proinflammatory state, microvasculopathy and fibrosis characteristic of SSc [9, 10].

The identification of circulating Tfh-like cells that are more accessible than Tfh cells in tissues has provided the opportunity to analyze these cells in autoimmune diseases [13]. Increased Tfh cell lineage activity and exaggerated Tfh cell response are more likely to be associated in the pathogenesis of autoimmune diseases. Tfh cells are involved in the disease pathogenesis of multiple sclerosis, myasthenia gravis, type 1 diabetes mellitus, autoimmune hepatitis, inflammatory bowel diseases and IgA nephropathy [13]. The association between disease activity and circulating Tfh cells was reported in patients with SLE [14]. Increased Tfh cells in the circulation and synovial fluid were found in RA patients with high DAS28 compared to moderate-low DAS28 [15]. An increased percentage of Tfh cells has been shown in the peripheral blood of patients with primary Sjögren’s syndrome with extra glandular findings [16]. However, there are limited data on the potential role of Tfh cells in SSc [17]. It was found that Tfh cells increased in SSc patients with extensive skin involvement compared to limited skin involvement [18]. In another study, Tfh-like cell infiltrates were found in skin lesions of patients with SSc and the infiltrates were positively associated with mRSS [19, 20]. CD4+ , ICOS+ and PD-1+ positive cell levels in SSc skin were demonstrated by immunohistochemistry and multicolor immunofluorescence staining, and a statistically significant correlation was demonstrated between mean mRSS [20]. However, there is limited information about peripheral Tfh cells and disease severity in SSc.

In this study, we aimed to investigate the association between follicular Tfh cells and disease severity in SSc, a chronic connective tissue disease characterized by progressive fibrosis.

## Materıal and methods

The study included fifty patients who were diagnosed with SSc based on the ACR/EULAR 2013 classification criteria [21]. Various demographic characteristics, including comorbid diseases such as hypertension and diabetes mellitus, as well as specific SSc-related factors like digital ulcer, pulmonary hypertension, lung involvement, sedimentation, C-reactive protein (CRP) levels and autoantibodies, were recorded for each patient. Disease activities were assessed using the modified Rodnan skin score (mRSS), which evaluated the thickness and extent of skin involvement through a scoring system ranging from 0 to 3 (mRSS = 0 normal skin, mRSS = 1 mild skin thickness, mRSS = 2 moderate skin thickness, mRSS = 3 severe skin thickness). Medsger’s disease severity scale was also utilized to evaluate the severity of SSc in nine organ systems, with a scoring system ranging from 0 to 4. The study protocol received approval from the faculty of medicine ethics committee and was designed in accordance with the principles outlined in the declaration of Helsinki (approval number: 53043469-050.04.04).

Flow cytometric analysis was performed on blood samples collected from the patients within two hours of collection. Fluorochrome-labeled monoclonal antibodies were used to analyze the numbers and percentages of Tfh cells (CD4+ CXCR5+), as well as the expressions of ICOS, CD40 and PD-1. Data analysis was conducted using the Kaluza flow cytometry analysis software. The absolute numbers of lymphocyte subtypes were calculated based on the absolute lymphocyte count obtained from flow cytometry and the percentage of lymphocyte subtypes.

Statistical analysis was performed using SPSS 21.0 software. Descriptive statistics, including mean ± standard deviation, median [25–75th percentile], frequency (*n*) and percentage (%), were used to summarize the data. The Kolmogorov–Smirnov test was employed to assess the normality distribution, and accordingly, the Mann–Whitney *U* test or independent sample *t *test was applied. The chi-square test was used for comparisons involving qualitative data. Spearman and Pearson correlation analyses were conducted to determine the presence and strength of linear relationships between numerical measurements. Correlation coefficients (*r*) were interpreted as very weak, weak, moderate, high or very high based on their values. A confidence interval of 95% was used, and a *p* value of less than 0.05 was considered statistically significant.

## Results

Fifty SSc patients (46 female and 4 male) were included in the study. The mean age was 59.2 ± 10.9 years, and the median follow-up was 9 years. 19 patients (38%) were limited cutaneous SSc (lcSSc) and 31 patients (62%) with diffuse cutaneous involvement (dcSSc). There were anti-centromere positivity in 11 patients and anti-Scl-70 positivity in 22 patients. The mean mRSS was higher in dcSSc. There was a statistically significant difference between the two groups. A total score of Medsger’s disease severity was 5.2 ± 2.3 in dcSSc and 4.0 ± 3.3 in lcSSc. There was no statistically significant difference between the two groups in terms of ESR, CRP and Medsger’s disease severity. Demographic and laboratory characteristics of patients with SSc are given in Table 1.

**Table 1:** Demographic and laboratory characteristics of patients with systemic sclerosis

	Diffuse cutaneous (*n* = 31)	Limited cutaneous (*n* = 19)
Age, year	57.5 ± 10.8	60.0 ± 10.8
Gender *n* (%)		
Female	27 (%87.1)	19 (%100)
Male	4 (%12.9)	–
Follow-up time, year	13.0 ± 10.9	9.2 ± 8.2
Comorbid disease, *n* (%)	18 (%58.1)	13 (%68.4)
ESR (mm/h)	27.6 ± 15.4	24.9 ± 20.7
CRP (mg/L)	2.6 [2.0–5.9]	2.0 [2.0–10.7]
Anti-centromere positivity, *n* (%)	1 (%3.8)	10 (%71.4)
Anti-Scl-70 positivity, *n* (%)	22 (%84.6)	–
Modified Rodnan skin score	18.1 ± 9.2	12.1 ± 7.7
Medsger disease severity score (total)	5.2 ± 2.3	4.0 ± 3.3

There was no statistically significant difference between the two groups in terms of CD4+ lymphocyte percentages. The CD4+ CXCR5+ lymphocyte percentages were 11.5% ± 5.6% in dcSSc and 9.8 ± 5.6% in lcSSc. The percentages of CD4+ ICOS+ , CD4+ CD40L+ and CD4+ PD1+ lymphocytes were similar between both SSc subgroups. There was no statistically significant difference between the groups in terms of the distribution of their expression (Table 2).

**Table 2:** Distribution of *T* lymphocyte expressions in systemic sclerosis

	Diffuse cutaneous	Limited cutaneous	*P* value
CD4+ CXCR5+	11.5 ± 5.6	9.8 ± 5.6	0.305
CD4+ ICOS+	5.3 ± 4.0	5.4 ± 2.4	0.954
CD4+ CD40L+	0.08 [0.0–0.3]	0.09 [0.0–0.2]	0.980
CD4+ PD1+	97.4 ± 2.8	96.4 ± 3.1	0.259

There was no significant correlation between CD4+ CXCR5+ , CD4+ CD40L+ , CD4+ ICOS+ and CD4+ PD1+ cell levels and inflammation markers. The mean CD4+ CXCR5+ *T* lymphocyte levels were higher in patients with Raynaud’s phenomenon, digital ulcer, heart involvement and lung involvement. In addition, both CD4+ CD40L+ and CD4+ PD1+ lymphocyte levels were higher in SSc patients with lung involvement compared to patients without lung involvement. However, no statistically significant was found. The distribution between clinical and laboratory findings and T lymphocyte expressions are shown in Table 3.

**Table 3:** Distribution between clinical and laboratory findings and T lymphocyte expressions in patients with systemic sclerosis

	CD4+ CXCR5+	CD4+ CD40L+	CD4+ ICOS+	CD4+ PD1+
Age, year	0.249	0.111	0.171	0.478
Gender *n* (%)				
Female	11.8 ± 4.9	0.09 [0–0.25]	2.8 ± 2.5	97.0 ± 3.0
Male	10.8 ± 5.7	0.02 [0–0.07]	5.6 ± 3.4	96.9 ± 1.1
Anti-centromere				
Positive	9.9 ± 6.0	0.08 [0–0.11]	4.9 ± 2.6	97.1 ± 2.7
Negative	10.8 ± 5.6	0.11 [0–0.33]	5.7 ± 4.0	97.2 ± 2.9
Anti-Scl-70				
Positive	11.2 ± 5.9	0.11 [0–0.30]	4.8 ± 2.1	97.8 ± 1.8
Negative	9.8 ± 5.5	0.08 [0–0.25]	6.3 ± 4.8	96.4 ± 3.6
Digital ulcer				
Positive	13.3 ± 6.8	0.08 [0–0.22]	5.4 ± 2.0	98.1 ± 1.8
Negative	10.4 ± 5.2	0.09 [0.02–0.33]	5.4 ± 3.7	96.7 ± 3.1
Pulmonary hypertension				
Yes	10.8 ± 6.3	0.12 [0.07–0.28]	5.1 ± 3.7	96.9 ± 1.8
No	10.9 ± 5.5	0.08 [0–0.21]	5.4 ± 3.4	97.0 ± 3.1
Lung involvement				
Yes	11.9 ± 5.3	0.08 [0–0.3]	5.2 ± 4.0	97.3 ± 2.8
No	9.4 ± 5.8	0.03 [0–0.18]	5.6 ± 2.5	96.5 ± 3.1

Considering the correlation between mRSS and T lymphocyte expressions, there was no significant correlation between mRSS and CD4+ CXCR5+ , mRSS and CD4+ CD40L+ , mRSS and CD4+ ICOS+ and mRSS and CD4+ PD1+ lymphocyte percentages. Considering the correlation between Medsger’s disease severity and T lymphocyte expressions, there was no statistically significant correlation between general, peripheral vascular, skin and heart severity and CD4+ CD40L+ , CD4+ ICOS+ , CD4+ PD-1+ T lymphocyte levels. A positive correlation was found between the severity of lung disease and CD4+ CXCR5+ T lymphocyte levels in patients with SSc (*p* = 0.01, *r* = 0.358). Correlations between T lymphocyte expressions and mRSS and Medsger disease severity are shown in Table 4.

**Table 4:** Correlation of T lymphocyte expressions with modified Rodnan skin score and Medsger disease severity score in patients with systemic sclerosis

	CD4+ CXCR5+	CD4+ CD40L+	CD4+ ICOS+	CD4+ PD1+
Follow-up time, year	0.058	0.099	0.083	0.043
ESR (mm/h)	0.045	0.110	0.146	0.058
CRP (mg/L)	0.161	0.032	0.198	0.137
Medsger disease severity score				
General	0.005	0.121	0.089	0.171
Peripheral vascular	0.082	0.116	0.065	0.053
Skin	0.001	0.061	0.053	0.177
Cardiac	0.020	0.074	0.060	0.104
Lung	0.358^*^	0.006	0.075	0.089
Medsger disease severity score	0.051	0.016	0.095	0.043

^*^p < 0.05

## Discussion

Tfh cells, a subset of CD4+ T cells, have been implicated in autoimmune diseases, including SSc. Previous studies have mainly investigated Tfh cells in peripheral blood, and their relationship with disease activity has been reported in systemic lupus erythematosus (SLE) [13, 14]. In our study, we examined Tfh cells in SSc patients and found higher percentages of Tfh cells in patients with specific clinical findings, such as digital ulcer, heart involvement and lung involvement. However, no significant correlation was observed between Tfh cell percentages and disease severity in terms of Medsger’s disease severity scale. We also assessed the relationship between Tfh cells and skin involvement, which is a characteristic feature of SSc.

Previous studies have reported Tfh-like cell infiltrates in skin lesions of SSc patients, and these infiltrates were positively associated with the severity of skin involvement [18, 22, 23]. It has been reported that circulating Tfh cells are increased in SSc compared to healthy controls [18]. The increase in Tfh cells was greater in the presence of diffuse SSc and arterial pulmonary hypertension. Also, circulating Tfh cells from patients with SSc had a higher capacity to differentiate into CD19+ CD27+ CD38hi B cells, in vitro [18]. In our study, Tfh cell percentages were higher in patients with extensive skin involvement compared to those with limited skin involvement, but no correlation was found between Tfh cell percentages and the mRSS, which measures skin sclerosis severity. In addition, no significant correlation was found between CD4+ CD40L+ lymphocyte level and total Medsger disease severity. Tfh-like cell infiltrates were found to be positively associated with mRSS [19, 20]. Also, CD4+ , ICOS+ and PD-1+ positive cell levels in SSc skin were demonstrated by immunohistochemistry and multicolor immunofluorescence staining and a statistically significant correlation was reported between mean mRSS [20]. In addition, CD4+ ICOS+ cells were also detected high in SSc skin [22]. In our study, the percentage of CD4+ PD1+ cells was higher in those SSc with extensive skin involvement than in SSc with limited skin involvement (97.4 ± 2.8 and 96.4 ± 3.1, respectively). However, no significant correlation was found between CD4+ PD1+ cell percentage and mRSS.

ICOS is involved in regulating humoral immune responses and has been proposed as a therapeutic target for fibroproliferative disorders [23, 24]. In SSc, higher serum levels of ICOS have been reported in patients with dcSSc [24]. However, in our study, no correlation was found between CD4+ ICOS+ cell percentages and skin sclerosis severity measured by mRSS and Medsger’s disease severity total score. Cell-to-cell adhesion molecules, JAM-A and JAM-C modulate cell adhesion, migration and neovascularization. In addition, JAM-A is an important mediator molecule that facilitates MM-associated angiogenesis [25]. The levels of these adhesion molecules levels correlate with many diseases’ severity [9–12]. The circulating levels of soluble JAM-A and sJAM-C were found to be significantly increased in early-stage SSc [9, 10]. And, no significant difference in sJAM-A and sJAM-C levels was found between lcSSc and dcSSc patients [9, 10]. However, we does not provide specific numbers or detailed analysis of the results about JAM-A and sJAM-C.

While this study contributes valuable insights into the relationship between Tfh cells and disease severity in SSc, certain limitations should be acknowledged. While this study contributes valuable insights into the relationship between Tfh cells and disease severity in SSc, there were a few limitations. Firstly, the sample size of fifty patients might be relatively small, potentially affecting the generalizability of the findings. Additionally, the study design was cross-sectional, limiting the ability to establish causality or determine the temporal association between Tfh cells and disease severity. Based on the limitations identified, further investigations are warranted to expand knowledge in this area. Increasing the sample size and conducting multi-center studies would enhance the generalizability of the findings. Longitudinal studies could help elucidate the temporal relationship between Tfh cells and disease severity in SSc. Moreover, exploring the interactions between Tfh cells and multiple organ systems affected by SSc would provide a more comprehensive understanding of the disease pathogenesis. Such studies may contribute to the development of targeted therapeutic strategies for SSc patients. A funding declaration is mandatory for publication in this journal. Please confirm that this declaration is accurate, or provide an alternative.
